# Myocarditis in CD8-Depleted SIV-Infected Rhesus Macaques after Short-Term Dual Therapy with Nucleoside and Nucleotide Reverse Transcriptase Inhibitors

**DOI:** 10.1371/journal.pone.0014429

**Published:** 2010-12-23

**Authors:** Lakshmanan Annamalai, Susan V. Westmoreland, Heber G. Domingues, Dennis G. Walsh, R. Gilberto Gonzalez, Shawn P. O'Neil

**Affiliations:** 1 Division of Comparative Pathology, New England Primate Research Center, Harvard Medical School, Southborough, Massachusetts, United States of America; 2 Neuroradiology, Martinos Center for Biomedical Imaging, Massachusetts General Hospital, Charlestown, Massachusetts, United States of America; University of California San Francisco, United States of America

## Abstract

**Background:**

Although highly active antiretroviral therapy (HAART) has dramatically reduced the morbidity and mortality associated with HIV infection, a number of antiretroviral toxicities have been described, including myocardial toxicity resulting from the use of nucleotide and nucleoside reverse transcriptase inhibitors (NRTIs). Current treatment guidelines recommend the use of HAART regimens containing two NRTIs for initial therapy of HIV-1 positive individuals; however, potential cardiotoxicity resulting from treatment with multiple NRTIs has not been addressed.

**Methodology/Principal Findings:**

We examined myocardial tissue from twelve CD8 lymphocyte-depleted adult rhesus macaques, including eight animals infected with simian immunodeficiency virus, four of which received combined antiretroviral therapy (CART) consisting of two NRTIs [(9-R-2-Phosphonomethoxypropyl Adenine) (PMPA) and (+/−)-beta-2′,3′-dideoxy-5-fluoro-3′-thiacytidine (RCV)] for 28 days. Multifocal infiltrates of mononuclear inflammatory cells were present in the myocardium of all macaques that received CART, but not untreated SIV-positive animals or SIV-negative controls. Macrophages were the predominant inflammatory cells within lesions, as shown by immunoreactivity for the macrophage markers Iba1 and CD68. Heart specimens from monkeys that received CART had significantly lower virus burdens than untreated animals (p<0.05), but significantly greater quantities of TNF-α mRNA than either SIV-positive untreated animals or uninfected controls (p<0.05). Interferon-γ (IFN-γ), IL-1β and CXCL11 mRNA were upregulated in heart tissue from SIV-positive monkeys, independent of antiretroviral treatment, but CXCL9 mRNA was only upregulated in heart tissue from macaques that received CART.

**Conclusions/Significance:**

These results suggest that short-term treatment with multiple NRTIs may be associated with myocarditis, and demonstrate that the CD8-depleted SIV-positive rhesus monkey is a useful model for studying the cardiotoxic effects of combined antiretroviral therapy in the setting of immunodeficiency virus infection.

## Introduction

Human immunodeficiency virus (HIV) infection has been associated with a variety of diseases of the cardiovascular system, including myocarditis [1], [2], [3], atherosclerosis and coronary heart disease [4], [5], [6], [7], [8], [9], [10], [11], and cardiomyopathy and ventricular dysfunction [12]. Myocarditis is the most common manifestation and is thought to result in dilated cardiomyopathy (2,4). Although HIV RNA, [13] DNA [14] and HIV-1 gp120 [15] have been localized infrequently in cardiomyocytes of HIV positive patients with myocarditis, the direct involvement of HIV infection and replication in the dysfunction of cardiomyocytes and in the pathogenesis of HIV-associated cardiac disease remains controversial. An indirect but more conspicuous role in the pathogenesis of HIV myocarditis and cardiomyopathy has been attributed to infected and/or immune activated inflammatory cells infiltrating the myocardium. Interestingly, myocarditis and dilated cardiomyopathy have also been reported in pathogenic simian immunodeficiency virus (SIV) infection of rhesus macaque monkeys [16], [17].

The prevalence of myocarditis since the advent of HAART has not been well defined; however, Pugliese and colleagues reported myocardial disease in 52% of 544 patients treated late in the course of AIDS with NRTIs versus 19% of 498 patients treated with HAART [18]. Although HAART has been credited with reducing virus burdens in plasma and lymphoid tissues, improving peripheral CD4^+^ T lymphocyte numbers and immune function and prolonging AIDS-free survival in HIV infected individuals [19], [20], cardiovascular disease and other complications that arise due to antiretroviral toxicity and immune reactivation continue to be problematic [21], [22], [23], [24]. Antiretroviral therapy has been associated with an increased risk of cardiovascular disease in HIV positive patients [7], [25], [26], although this remains controversial [8], [10], [27]. Myocardial toxicity resulting from treatment with NRTIs and non-nucleoside reverse transcriptase inhibitors (NNRTIs) has been reported previously; suspected mechanisms include mitochondrial toxicity and the formation of reactive oxygen species (ROS) within cardiac myocytes [28], [29] [29], [30], [31], [32], [33]. Furthermore, chronic exposure to NRTIs, NNRTIs, or protease inhibitors (PIs) can cause oxidative stress to endothelial cells, resulting in enhanced cytokine production and recruitment of mononuclear cells [34].

During routine histopathological examination, myocarditis was observed in four of four CD8^+^ lymphocyte-depleted rhesus macaques that had been infected with SIVmac251 and treated with the NRTIs PMPA and RCV for 28 days. In contrast, myocarditis was not evident in CD8-depleted, SIV-infected macaques that did not receive CART, suggesting possible antiretroviral agent-mediated myocardial toxicity. The present study was conducted to investigate the pathogenesis of myocarditis in these animals.

## Methods

### Ethic Statement

All animal studies were performed in accordance with federal laws and regulations, international accreditation standards, and institutional policies, including approval by the New England Primate Research Center (NEPRC), Harvard Medical School Animal Care and Use Committee of Harvard University (IACUC protocol # 03076). All animals received environmental enrichment and were monitored daily for evidence of disease and changes in attitude, appetite, or behavior suggestive of illness. Appropriate clinical support was administered under the direction of the attending veterinarian and included analgesics, antibiotics, intravenous fluids, and other supportive care. Steps were taken to minimize suffering and discomfort. Animals were pair or group-housed when possible. Surgical and sampling procedures were kept to a minimum and were conducted under anesthesia followed by appropriate analgesics for pain and discomfort, which was carefully monitored. Animals were euthanized under anesthesia when they presented with advanced stages of AIDS; criteria for euthanasia included 15% weight loss in two weeks, unresponsive opportunistic infection, persistent anorexia, intractable diarrhea, progressive neurologic signs, significant cardiac or pulmonary signs or other serious illness.

### Tissues

This retrospective study used archived frozen left ventricles that had been collected during the necropsy of CD8 lymphocyte-depleted rhesus macaques (control group; n = 4), CD8 lymphocyte-depleted and SIVmac251-infected rhesus macaques with simian AIDS (untreated group; n = 4), and CD8 lymphocyte-depleted and SIVmac251-infected rhesus macaques with AIDS that had been treated with a combined antiretroviral regimen (CART group; n = 4) (**Table 1**). The CART regimen consisted of 28 days of therapy with PMPA (9-R-2-Phosphonomethoxypropyl Adenine), at 30 mg/kg/day and Racivir (RCV, (+/−)-beta-2′,3′-dideoxy-5-fluoro-3′-thiacytidine), at 10 mg/kg/day, initiated 28 days after infection with SIV, and administered as once-daily subcutaneous injections, as described elsewhere [35]. Portions of left ventricle collected at necropsy were immediately immersed in RNAlater solution (Qiagen Inc., Valentia, CA), held at 4°C overnight, and frozen the following day at –80°C, according to the manufacturer's recommendations. Histopathologic examination was performed on hematoxylin and eosin (H&E) stained sections of cardiac tissue that were fixed in 10% neutral buffered formalin before routine processing and embedding in paraffin.

**Table 1:** Animals and Therapy.

Group	Case number	Age (days)	DAD (days)	CART (days)	AIDS	Plasma virus load (log_10_ copies/ml)		Myocarditis
						21 dpi	terminal	
Control	Case 1	1668	-	No	-			-
	Case 2	1549	-	No	-			-
	Case 3	1794	-	No	-			-
	Case 4	1726	-	No	-			-
Untreated	Case 5	3116	70	No	Yes	7.7	6.7	-
	Case 6	3487	57	No	Yes	7.7	7.6	-
	Case 7	3489	63	No	Yes	7.1	7.7	-
	Case 8	4606	85	No	Yes	6.6	7.6	-
CART	Case 9	4025	57	28	No	7.4	5.4	++
	Case 10	7584	57	28	No	7.1	6.1	+++
	Case 11	3782	57	28	No	6.5	4.6	++
	Case 12	3778	57	28	No	7.2	5.8	++

DAD: duration of infection in days.

### Immunohistochemistry

Immunohistochemistry (IHC) was used to characterize the phenotype of immune cells in myocardial lesions and to localize chemokines, proinflammatory cytokines, and adhesion molecules in sections of myocardium. The antibodies used were directed against macrophages (Iba-1, Waco, Richmond, VA), activated macrophages (CD68, clone KP-1, Dako Cytomation, Carpinteria, CA), T lymphocytes (CD3, Dako), CD4^+^ cells (CD4, clone 1F6, Vector Laboratories, Burlingame, CA), CD8^+^ cells (CD8, clone 1A5, Vector Labs), B lymphocytes (CD20, clone L26, Dako), the inactive precursor of IL-18 (pro-IL-18, R&D Systems, Minneapolis, MN), the chemokine CXCL9 (also known as monokine induced by gamma interferon, or MIG, R&D Systems), the adhesion molecule ICAM-1 (CD54, clone 54CO4, LabVision, Fremont, CA) and cytomegalovirus (CMV) (rabbit anti-CMV serum, a gift from Peter A. Barry, California National Primate Research Center, University of California at Davis). IHC was performed on 5 µM sections of formalin-fixed, paraffin-embedded (FFPE) tissues, using an ABC immunoperoxidase technique as described elsewhere [36]. Briefly, FFPE tissue sections were deparaffinized in xylene and rehydrated through graded ethanol to distilled water. Microwave antigen retrieval was performed using citrate buffer (Dako Corp., Carpinteria, CA) for all antibodies except CD8, for which antigen retrieval was accomplished using a pressure cooker and Trilogy solution (Cell Marque, Rocklin, CA). Endogenous peroxidase activity was blocked in 3% hydrogen peroxide in phosphate buffered saline (PBS), and non-specific protein binding was blocked with Protein Block (Dako). After incubating with the primary antibody, tissue sections were reacted sequentially with biotinylated secondary antibody (Dako), horseradish peroxidase-conjugated streptavidin (Dako), and the chromogenic substrate 3, 3′-diaminobenzidene (DAB, Dako), and counterstained with hematoxylin (Sigma Chemical Co., St. Louis, MO). The source and dilution of antibodies used in this study are summarized in **Table 2**.

**Table 2:** Antibodies used for immunohistochemistry.

Antigen	Source	Antibody type*	Dilution
CD3	Dako	Polyclonal	1∶600
CD4	Vector	IgG1	1∶40
CD8	Vector	IgG1	1∶50
CD20	Dako	IgG2a	1∶175
CD68	Dako	IgG1	1∶410
Iba-1	Waco	Polyclonal	1∶1,000
ICAM-1	Labvision	IgG1	1∶100
CXCL9/MIG	R&D Systems	IgG	1∶100
IL-18-pro	R&D Systems	IgG	1∶100
CMV	Non commercial	Polyclonal	1∶1600

*CXCL9/MIG and IL-18-pro antibodies were raised in goat; CD3 and Iba 1 polyclonal antibodies were raised in rabbit.

### Double immunolabeling and spectral imaging

Double label immunohistochemistry was performed on deparaffinized and rehydrated formalin-fixed paraffin embedded tissue sections by sequentially incubating with the first primary antibody and its corresponding secondary antibody followed by detection of antigen-antibody complex using the Fast Red Substrate-Chromogen System (Dako Cytomation) catalyzed by ABC-Alkaline Phosphatase (Vectastain ABC-AP Standard Kit, Vector). The tissue sections were subsequently incubated with the second primary antibody and respective secondary antibody. The second immune complex was visualized by DAB chromogen (DakoCytomation) as in single immunohistochemistry and the tissue sections were counterstained with hematoxylin. Images of labeled sections were acquired using an Olympus Vanox-S AHBS microscope interfaced with a liquid crystal tunable filter based camera (CRI, Woburn MA, USA) from 420 nm to 720 nm at 20 nm steps. The spectral components were unmixed using single stain controls as described elsewhere [17], [37]. Spectrally unmixed individual images were then pseudo-colored and combined.

### Masson's trichrome stain

The extent of myocardial fibrosis was assessed by labeling collagen fibers in myocardial sections by the Masson's trichrome technique (Trichrome Stain, Newcomer supply, Middleton, WI). Briefly, FFPE tissue sections were deparaffinized in xylene, rehydrated in graded ethanol and post-fixed in Bouin's fixative for one hour at 55°C. The nuclei and collagen were sequentially stained with equal volumes of ferric chloride solution and alcoholic hematoxylin and trichrome solution, respectively.

### *In situ* hybridization (ISH)

Productively infected cells were identified in FFPE myocardial tissue sections by ISH for SIV RNA, as described elsewhere [36]. Tissue sections were deparaffinized in xylene and rehydrated in graded ethanol to diethyl pyrocarbonate (Sigma) treated water. Endogenous alkaline phosphatase activity was blocked with levamisole (Sigma), and sections were hydrolyzed in HCl (Sigma), digested with proteinase K (Roche Diagnostics Corp., Indianapolis, IN), acetylated in acetic anhydride (Sigma), and hybridized overnight at 50°C with a digoxigenin-labeled antisense riboprobe which spans the entire genome of the SIVmac239 molecular clone of SIVmac251 (Lofstrand Labs, Gaithersburg, MD). The following day, tissue sections were washed extensively and bound probe was detected by IHC, using alkaline phosphatase-conjugated sheep anti-digoxigenin F(ab) fragments (Roche) and the chromogen nitroblue tetrazolium/5-bromo-4-chloro-3-indolyl-phosphate (NBT/BCIP, Roche). Sections were counterstained with nuclear fast red (Vector Labs). Sections of brain from a rhesus macaque with SIVmac251 encephalitis served as both positive control (when incubated with SIV antisense probe) and negative control (when reacted with SIV sense probe). Additional negative controls included sections of myocardial tissue from uninfected macaques reacted with SIV antisense probe.

### Total RNA isolation and quantification

Total RNA was isolated from frozen left ventricle specimens. Briefly, 75-100 mg of tissue was homogenized in 1.5 ml of Trizol reagent (Invitrogen Corp., Carlsbad, CA) using a bead beater and 1 mm diameter silica beads (Biospec Products Inc., Bartlesville, OK). After homogenization, the homogenate was removed to a new tube, 0.2 volumes of chloroform was added, and the aqueous phase was collected after centrifugation at 8,000× g for 5 minutes at 4°C. The aqueous phase was combined with an equal volume of 70% ethanol, one ml of 4M guanidinium isothiocyanate was added, and the mixture was loaded onto a RNeasy spin column (Qiagen). After on-column DNase treatment (Qiagen), the total RNA was eluted in RNase free water, 2 units/µl of recombinant RNase inhibitor (RNasin, Promega) was added, and the RNA was frozen at –80°C. The amount of RNA extracted from tissue specimens was measured using the Quant-iT Ribo Green RNA assay (Invitrogen), adhering to the manufacturer's instructions, and measuring fluorescence for 0.1 seconds at excitations and emissions of 485 nm and 535 nm, respectively, using a Victor^3^ V 1420 Multilabel Counter (Perkin Elmer, Waltham, MA).

### TaqMan real time RT-PCR

The absolute quantification of SIV Gag, TNF-α and RPL13A transcripts in total RNA from left ventricle and spleen specimens was conducted using TaqMan probes and TaqMan One-Step RT-PCR master mix reagents (Applied Biosystems, Foster City, CA) on an ABI Prism 7700 thermal cycler (Applied Biosystems). Total RNA from myocardial tissues (100 ng per specimen) was combined with 200 nM each of forward and reverse primers, 1x One-Step RT-PCR master mix and, 100 nM of TaqMan probe in a 50 µl reaction. For use as internal RNA standards, known copy numbers of RNA transcripts were serially diluted in RNase free water from 10^6^ through 10^1^ copies. Amplification data were analyzed using Sequence Detection Version 1 software (Applied Biosystems).

### SYBR Green real time PCR

To investigate the expression of cytokines and chemokines in myocardial tissue, 20 ng of cDNA from each specimen was used as template in a 25 µl reaction containing 1x SYBR Green master mix (Applied Biosystems) and forward and reverse primers (100 mM each). Reactions were conducted in duplicate on an ABI 7500 thermal cycler, with the following conditions: 1 cycle of 96°C for 2 minutes; 45 cycles of 96°C for 30 seconds and 60°C for 1 minute followed by one cycle of dissociation from 96°C to 60°C. At the end of each run, the data were analyzed using Sequence Detection Version 1 software (Applied Biosystems). Fold-regulation of expression of each gene was calculated from the Ct values after normalizing with the Ct values obtained for RPL13A mRNA, the internal control gene. The primers used in the study are shown in **Table 3**.

**Table 3:** Primers and probes.

Accession Number	Gene	Primer
**Primers and probes used for TaqMan assay**		
M33262	SIV gag	Forward: AGAAAGCCTGTTGGAGAACAAAGAAGGReverse: AGTGTGTTTCACTTTCTCTTCTGCGTGProbe: 6FAM CTGTCTGCCTCATCTGGTGC TAMRA
NM_001047149	TNF-α	Forward: CTCTTCAAGGGCCAAGGCTReverse: GATGCGGCTGATGGTGTGProbe: 6FAMCCCCTCCAACCATGTGCTCCTCA TAMRA
FM208094	RPL13A	Forward: CGAGAAAGTTTGCCTATCTGGG*Reverse: GGTGGTTGTCACTGCCTGGTA*Probe: 6FAM CCTGGCTCACGAGGTTGGCTGG TAMRA
**Primers used for SYBR Green assay**		
U19845	IL-1β	Forward: TGGCATCCAGCTACAAATCTCCCAReverse: AAGGGAATCAAGGTGCTCAGGTCA
NM_001042733	IL-6	Forward: AGCCACTGACCTCTTCAGAACGAAReverse: GTGCCTCTTTGCTGCTTTCACACA
NM_001044727	IL-10	Forward: TCCTTGCTGGAGGACTTTAAGGGTReverse: TCACATGCTCCTTGATGTCTGGGT
NM_001032834	IL-18	Forward: TTCATTGACCAAGGAAATCGGCCCReverse: GCCATACCTCTAGGCTGGCTATCTTT
NM_001032905	IFN-γ	Forward: GACTCGAATGTCCAACGCAAAGCAReverse: TCGACCTCGAAACATCTGACTCCT
NM_001032936	CXCL9	Forward: TGGGCATCATCTTCCTGGTTCTGAReverse: TTTCTCGCAGGAAAGGTTTGGAGC
NM_001032950	CXCL11	Forward: TGTCTTTGCATAGGCCCTGGAGTAReverse: GCTTGCTTCGATTTGGGATTTAGGC
NM_001047135	ICAM-1	Forward: TCCGTCAAAGTGAATTGCAGTGCCReverse: CCCATCAGGGCAGTTTGAATAGCA

Note:

* RPL13A primer set was used for both TaqMan and SYBR Green assays.

### Statistical analysis

A two-tailed, nonparametric Mann-Whitney U test was used to compare the copy numbers of SIV and TNF-α mRNA transcripts, which are reported as median values ± semi-interquartile range. Significant differences were assumed for probability values of *p*<0.05.

## Results

### Histopathology

**Figure 1:**
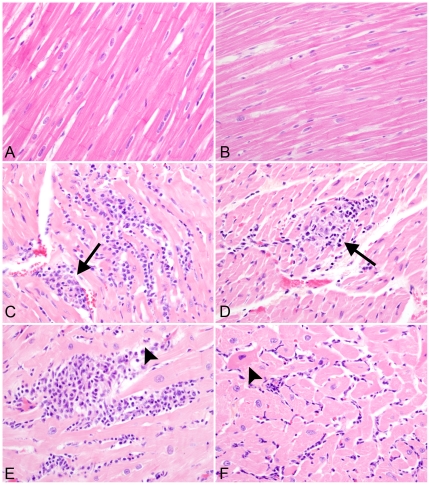
Myocardial lesions are present in all four monkeys that receive CART. H & E stained sections of left ventricle from CD8^+^ cell-depleted, uninfected control macaque (A) and SIV-positive untreated macaque (B), showing normal cardiomyocytes with cross striations. In contrast, sections of myocardium from monkeys that received CART contained multifocal infiltrates of mononuclear inflammatory cells that separated cardiomyocytes (C), cardiomyocytolysis and necrosis (arrow heads in D and E) and hypereosinophilia with loss of cross striations in cardiomyocytes (arrow in F). Hematoxylin & eosin stain. (400X).

**Figure 2:**
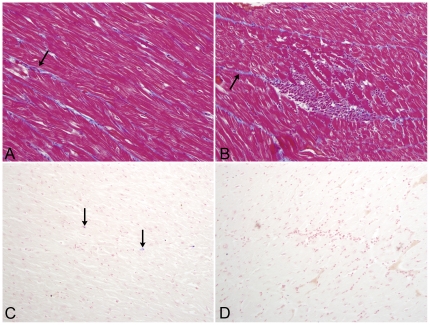
Masson's trichrome staining and *In situ* hybridization. Masson's trichrome stain was performed to localize collagen fibers (blue stain, indicated by arrow) within myocardial sections of both control animals and animals that received CART. Significant fibrosis was not apparent in any animals from either group (200X). Small numbers of infected cells (blue chromogen) were localized by in situ hybridization for SIV RNA in myocardial sections from untreated, SIV-infected control macaques (C) and monkeys that received CART (D). NBT/BCIP chromogen (blue) with nuclear fast red counterstain (100X).

Histopathological examination of routine H&E sections revealed multifocal infiltrates of mononuclear inflammatory cells (morphologically consistent with macrophages and lymphocytes), which were dissecting and compressing cardiomyocytes in sections of myocardium from all four animals in the CART group. Necrosis and degeneration of cardiomyocytes was apparent within the lesions, with no evidence of fibrosis (**Figure 1**). In addition, reactive, hypertrophic endothelial cells were observed bulging into vascular lumens throughout the myocardial sections of 3 of the 4 animals that received CART. In contrast, no degenerative changes or significant inflammatory foci were present in the myocardial sections from any animals in either the control group or the untreated SIV positive animals with simian AIDS. The absence of intralesional fibrosis in the myocardium of affected animals was confirmed by Masson's trichrome stain, which revealed that there was no significant difference in the amount of collagen present within the myocardium of animals among all three groups (**Figures 2A and 2B**). The pericardium was normal in all animals.

### Myocardial lesions are composed of macrophages and lymphocytes

**Figure 3:**
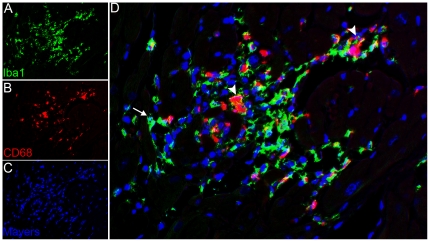
Activated macrophages are localized within inflammatory foci. Multiparameter immunohistochemistry and multispectral imaging to localize the macrophage marker Iba-1 (green) and the activated macrophage marker CD68 (red). Double immunolabeling and spectral imaging with pseudofluorescence coloring identifies Iba-1 expressing macrophages (green) within inflammatory foci (A). Expression of CD68 (red) identifies activated macrophages within inflammatory foci (B). Nuclei are stained with Mayer's hematoxylin counterstain (blue) (C). Note co-localization of all CD68 expression within Iba-1 expressing cells (D) (arrows) (400X).

**Figure 4:**
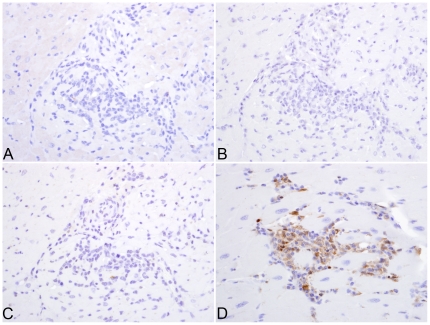
CD8^+^ T- and CD20^+^ B-lymphocytes are not localized within inflammatory foci. Immunohistochemistry using anti-CD20 identified a few B-lymphocytes in the inflammatory foci (arrows) (A). In contrast, no CD8^+^ T-lymphocytes are localized using anti-CD8 antibody (B) (400X). Immunohistrochemistry using anti-pro-IL-18 (C) or anti-CXCL9 (D) reveals the expression of these molecules on mononuclear cells within inflammatory foci (200X).

**Figure 5:**
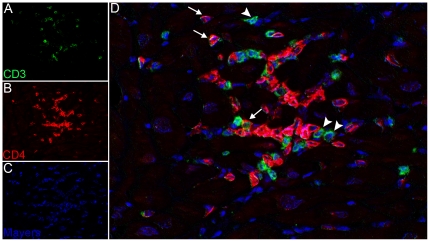
Lesions are composed of lymphocytes and macrophages. Double immunolabeling and spectral imaging with pseudofluorescence coloring identifies CD4^+^ T-lymphocytes and macrophages (red) within inflammatory foci (B). Expression of CD3 (green) identifies lymphocytes within inflammatory foci (A). Nuclei are stained with Mayer's hematoxylin (blue) (C). CD4^+^ lymphocytes express both CD4 and CD3 molecules (arrows) (D). Note CD3 expression on cells that do not express CD4 (arrowheads).

IHC was used to characterize the inflammatory infiltrates in the myocardium. Inflammatory foci were composed largely of Iba-1 expressing cells, compatible with macrophage lineage; moreover, 60–80% of the Iba-1 positive cells also expressed the CD68 glycoprotein, suggestive of an activated phenotype **(** **Figure 3** **)**. IHC failed to reveal significant expression of CD8 (which is expressed by both CD8^+^ T lymphocytes and natural killer (NK) cells) or CD20 (which identifies B lymphocytes) within inflammatory foci (**Figures 4A and 4B**). Because small numbers of CD3^+^ cells had been localized within the inflammatory foci by single-label IHC in the absence of significant CD8 expression, multiparameter IHC and spectral imaging were used to definitively identify the phenotype of intralesional T lymphocytes. Dual-label IHC for CD3 and CD4 expression revealed small numbers of CD3^+^/CD4^+^ double-positive lymphocytes and less frequent CD3^+^/CD4^-^ cells, which were assumed to be CD3^+^/CD8^+^ T lymphocytes (**Figure 5**). Taken together, these results indicate that the inflammatory foci were primarily composed of activated macrophages and small numbers of CD4^+^ positive helper T lymphocytes. Despite the abundance of activated macrophages in the myocardial lesions, expression of pro-IL-18, the inactive precursor of the proinflammatory cytokine IL-18, was confined to faint cytoplasmic reactivity in a minority of the macrophages present in the inflammatory foci (**Figures 4C and 4D**).

### Virus burden is significantly lower in the myocardium of animals that received CART

**Figure 6:**
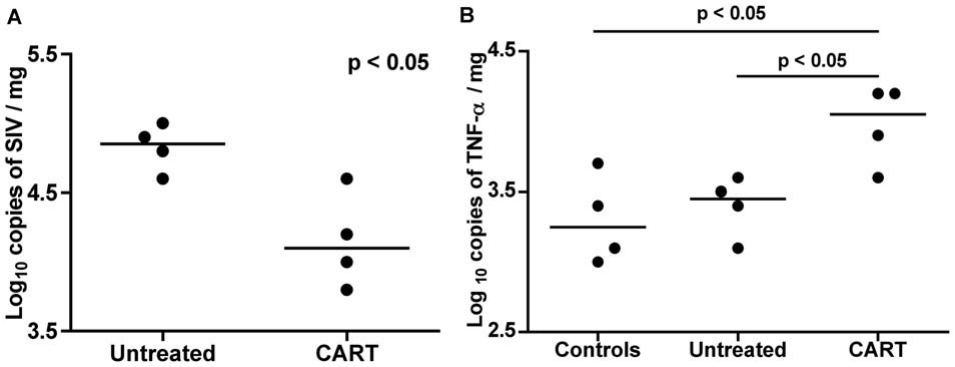
Quantification of virus burden and expression of TNF-α in myocardial tissue by real time RT-PCR. Tissue virus burdens were significantly lower in the left ventricles of animals that received CART as compared to untreated controls (**A**). Horizontal bars represent median values for each data set. Real-time RT-PCR with TNF-α specific TaqMan probe revealed significantly greater levels of TNF-α expression in the myocardium of animals that received CART as compared to control animals (**B**). Note that TNF-α expression in the untreated group is similar to SIV-negative controls. Horizontal bars represent median values for each data set.

ISH for SIVmac239 RNA revealed very few productively infected cells in the myocardium of either untreated animals or those that received CART (**Figures 2C and 2D**). However, quantification of viral RNA by TaqMan real time RT-PCR revealed a significantly lower virus burden in the myocardium of animals that received CART as compared to the myocardium of untreated animals (median values of 4.10±0.18 versus 4.85±0.09 log_10_ SIV copies/mg, respectively; p<0.05) **(** **Figure 6A** **)**. These results indicate that the myocardium is not a primary target organ for virus replication in CD8 lymphocyte-depleted animals, and suggest that the lesions present in the myocardium of animals in the CART group are not directly associated with SIV infection. In addition to lower myocardial tissue virus burden, CART was associated with a significantly lower terminal plasma virus load (**Table 1**).

### Enhanced expression of the pro-inflammatory cytokine TNF-α in the myocardium of animals that received CART

We quantified mRNA transcripts for the proinflammatory cytokine TNF-α in myocardial tissue from all animals in the control, untreated and CART groups. Significantly greater levels of TNF-α mRNA expression were measured in the myocardium of animals that received CART than in myocardial tissue from the untreated or control macaques (4.05±0.19 vs. 3.45±0.10 vs. 3.25±0.20 log_10_ TNF-α copies/mg, respectively; *p*<0.05) (**Figure 6B**). There was no difference in the quantity of TNF-α mRNA transcripts measured in myocardial tissue from untreated versus control groups.

### Higher levels of CXCL9 (monokine induced by gamma interferon) expression in the myocardium of animals that received CART

Since macrophages were the principal cellular component of the inflammatory and degenerative myocardial lesions observed in animals that received CART, and significantly higher levels of TNF-α mRNA expression had been measured in myocardial specimens from the CART group, we sought to measure the levels of mRNA expression for other key proinflammatory cytokines and chemokines that have been implicated in the pathogenesis and regulation of cytotoxic injury. To that end, we used SYBR Green real time RT-PCR and gene specific primers to measure the quantity of several mRNA transcripts, including IL-1β, IL-6, IL-10, IL-18, INF-γ, MCP-1, CXCL9 and CXCL11 in frozen specimens of myocardial tissue from all three groups of animals. Myocardial tissue from control animals expressed very low levels of IL-1β, Il-6, IL-10, IL-18, CCR5 and INF-γ mRNA, with cycle threshold (Ct) values ranging from 33-37 (data not shown). In contrast, appreciable levels of MCP-1, CXCL9, CXCL11 and ICAM-1 mRNA were detected in controls, with Ct values ranging from 27-31, indicating an abundance of expression of these genes in the myocardium. **Table 4** shows the expression patterns of cytokines in the myocardium from untreated and CART groups of SIV-positive monkeys as compared to the SIV-negative CD8-depleted control group. Higher levels of CCR5, CXCL11, INF-γ, and IL-1β mRNA were expressed in the myocardium of SIV-positive animals, regardless of whether they received antiretroviral therapy, although the quantity of CXCL11 mRNA expressed in SIV-infected, untreated macaques was more than two-fold greater than in SIV-positive animals that received CART. The level of IL-18 mRNA expression was lower in both treated and untreated SIV-positive macaques, while IL-6 mRNA levels were similar to SIV-negative controls. Interestingly, while the quantities of IL-10, ICAM-1 and MCP-1 mRNA were similar in myocardial specimens from SIV-positive macaques that received CART and SIV-negative controls, greater quantities of all three transcripts were measured in myocardial specimens from SIV-infected, untreated animals than in the other two groups. In contrast, while myocardial CXCL9 mRNA levels were similar between SIV-negative animals and SIV-positive monkeys that received CART, the quantity of myocardial CXCL9 transcripts was 7-fold lower in untreated SIV-infected animals.

**Table 4:** Cytokine expression analysis.

Gene	Untreated	CART
IL-1β	↑ 4.9502	↑ 4.6028
IL-6	1.7261	1.2946
IL-10	↑ 3.2378	1.4871
IL-18	↓ −3.4702	↓ −3.2378
INF-γ	↑ 5.1248	↑ 5.8361
CXCL9	↓ −7.1354	1.3637
CXCL11	↑ 6.0945	↑ 2.7132
ICAM-1	↑ 4.0208	1.8693
MCP-1	↑ 2.2423	1.5263
CCR5	↑ 3.7451	↑ 3.1383

**Figure 7:**
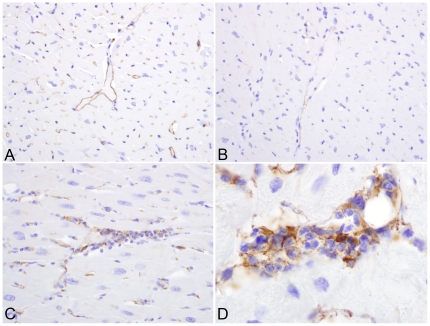
ICAM-1 expression in myocardial sections. IHC for ICAM-1, localizing increased ICAM-1 expression to endothelial cells in non-inflamed myocardium of SIV positive, untreated rhesus macaques (A) as opposed to SIV infected monkeys that received CART (B). Increased ICAM-1 expression was also localized to mononuclear inflammatory cell infiltrates in animals that received antiretroviral therapy (C). Higher magnification of inflammatory foci (1,000X) (D). Immunoperoxidase immunohistochemistry, with DAB chromogen (brown) and hematoxylin counterstain.

With regard to the relative magnitude of CXCL9 and ICAM-1 mRNA expression, differences between treated and untreated SIV-positive macaques correlated precisely with the pattern of protein expression in tissue sections by immunohistochemistry. Strong expression of CXCL9 (which is induced by IFN-γ) was observed by the macrophages present in the inflammatory foci (**Figures 4B, 4C and 4D**) in the myocardium of animals that received CART, while CXCL9 expression was not detected in sections of myocardium from untreated monkeys. Surprisingly, the expression of ICAM-1, which is induced by TNF-α, was significantly weaker in myocardial sections from animals that received CART as opposed to SIV-infected, untreated monkeys (**Figure 7**).

## Discussion

The independent and combined contributions of HIV infection and antiretroviral therapy on myocardial disease remain unclear; thus, a reliable animal model would benefit efforts to distinguish the impact of viral pathogenesis from antiretroviral toxicity. In this retrospective study, we investigated the pathogenesis of myocarditis in four of four CD8^+^ cell-depleted, SIV-infected rhesus macaques that had been treated for 28 days with PMPA and RCV. Several potential etiologies could account for myocarditis in these animals, including SIV infection, infection with opportunistic agents, immune reconstitution inflammatory syndrome (IRIS), autoimmunity, and antiretroviral toxicity. As described elsewhere, CD3^+^/CD8^+^ T lymphocytes were persistently depleted from the peripheral circulation in all SIV-positive animals for a minimum of 28 days post treatment with the CD8-depleting antibody, cM-T807; however, peripheral CD8^+^ T cells were partially restored in all depleted animals prior to necropsy [35].

All untreated SIV-infected animals were euthanized due to the development of simian AIDS and had AIDS defining lesions at necropsy; however, there were no significant inflammatory or degenerative changes present in the myocardium of these animals. The animals that received CART were electively sacrificed at 57 dpi, and were not clinically ill when euthanized, nor did they have lesions indicative of simian AIDS at necropsy. Sections of myocardium from animals that received CART contained infiltrates of mononuclear inflammatory cells with cardiomyocytolysis and cardiomyocyte atrophy. SIV has been associated with myocarditis, and in severe cases, dilated cardiomyopathy in chronically infected rhesus macaques [16], [17]. The inflammatory infiltrates that have been described previously for SIV-associated myocarditis are similar to those of HIV myocarditis, and are composed predominantly of CD8^+^ T lymphocytes with variable numbers of productively infected, SIV-positive macrophages and multinucleated giant cells [16], [17]. In our study, inflammatory foci within the myocardium were dominated by macrophages, and contained smaller numbers of CD3^+^/CD4^+^ T lymphocytes, but no multinucleated giant cells, CD8^+^ lymphocytes, or CD20^+^ B lymphocytes. The heart was not a primary target organ for SIV in these CD8^+^ cell-depleted animals, as very few SIV^+^ cells were localized in the myocardium of CART or untreated monkeys by ISH, and significantly fewer copies of SIV RNA were measured by real time RT-PCR in the myocardium of animals that received CART as compared to untreated animals. Taken together, these data provide strong evidence that the myocarditis present in the CART group of animals is not due to SIV infection or an effect of CD8^+^ T lymphocyte depletion. The presence of ongoing cardiomyocyte necrosis and the absence of myocardial fibrosis in the CART group suggests that the myocardial lesions are active but not chronic; however, we were unable to localize the temporal onset of myocarditis in these animals, as neither functional cardiovascular tests nor serum biochemical analyses specific to the cardiovascular system were carried out during the course of the study.

Both HIV-associated cardiomyopathy and chronic heart failure have been associated with activation of the immune system leading to the pathogenic overproduction of several proinflammatory cytokines, including TNF-α, IL-1β, IL-6 and IL-18 [38], [39]. Elevated local cardiac production and increased circulating levels of TNF-α [40], [41], [42] have been implicated in cardiomyopathies of various etiologies [43], [44]. Yearley and colleagues recently reported a direct relationship between dilated cardiomyopathy and TNF-α expression in SIV-infected rhesus macaques [45]. In this study, significantly greater levels of TNFα mRNA were expressed in the myocardial tissue from the CART group compared to the uninfected controls and SIV-infected untreated animals, whereas there was no difference in the quantity of TNF-α mRNA transcripts measured in spleen specimens from both untreated macaques and those that received CART (data not shown), suggesting similar systemic levels of TNF-α expression among SIV infected animals. The effects of TNF-α expression in the heart may have been augmented by increased myocardial IL-1β production in SIV infected macaques, as the quantity of IL-1β mRNA transcripts was elevated several fold in both the untreated and CART groups of animals. We also found three-fold higher expression of IL-10 mRNA in the untreated animals, which may have diminished the synergistic effects of TNF-α and IL-1β in the untreated group through its anti-inflammatory effects [46], [47]. Taken together, these data strongly suggest that locally produced myocardial TNF-α played a significant role in the pathogenesis of the myocarditis that was observed selectively in monkeys that received CART.

Interferon-gamma (IFN-γ) is a pleiotropic cytokine that plays important roles in both innate and adaptive, cell-mediated immunity. The role for IFN-γ is not as well established in the pathogenesis of myocardial disease; however, transgenic mice that constitutively express IFN-γ in liver tissue driven by the liver specific promoter of human serum amyloid P component (SAP) gene were recently shown to develop chronic active myocarditis and cardiomyopathy [48]. We measured mRNA transcripts for IFN-γ and the IFN-γ-inducible T cell-chemoattractant genes CXCL11 (I-TAC) and CXCL9 (MIG) in the myocardium from all three groups of monkeys. Compared to control macaques, six- and three-fold higher quantities of CXCL11 mRNA were expressed in the myocardial tissue of the SIV-infected untreated and CART groups, respectively. In contrast, seven-fold lower levels of CXCL9 mRNA were measured in the myocardium of untreated macaques compared to the control and CART groups. These results correlated favorably with the levels of CXCL9 protein expression observed by IHC, as higher levels of CXCL9 were localized in myocardial endothelial cells and intralesional macrophages in the myocardium of animals that received CART than in untreated macaques. Since TNF-α and IFN-γ can increase endothelial CXCL9 expression [49], increased TNF-α and IFN-γ mRNA expression may have induced CXCL9 expression and facilitated myocarditis in the macaques that received CART.

Despite the presence of myocarditis, macaques in the CART group expressed lower quantities of MCP-1 and ICAM-1 mRNA in myocardial tissue compared to untreated animals. It is unclear why the MCP-1 mRNA levels were lower in animals with lymphohistiocytic inflammation. However, in the case of ICAM-1, this unexpected result was explained by IHC, which revealed significant diffuse ICAM-1 expression in SIV-infected untreated animals, which was localized to endothelial cells in the non-inflamed myocardium. In contrast, in infected animals that received CART, ICAM-1 expression was confined to inflammatory foci. Since HIV gp120 has been shown to increase endothelial expression of ICAM-1 in vitro [50], we hypothesize that SIV-infection resulted in diffuse upregulation of ICAM-1 expression by myocardial endothelial cells.

Elevated levels of IL-18 mRNA have been measured in the myocardium of mice after the induction of myocardial infarction [51], while both increased [52] and decreased [53] IL-18 mRNA expression have been reported in myocardial tissue from human patients with ischemic and dilated cardiomyopathy. Surprisingly, despite increased expression of TNF-α and IL-1β mRNA, we observed a three-fold decrease in IL-18 mRNA expression in myocardial tissue from monkeys in the CART group as compared to controls. However, since IL-18 is regulated post-transcriptionally by caspase-1 and IL-18 binding protein, mRNA levels are not necessarily predictive of IL-18 activity [54], [55]. In the present study, IHC using an anti-pro-IL-18 antibody resulted in faint intracytoplasmic reactivity of macrophages within inflammatory foci; however, the expression of mature IL-18 protein could not be evaluated due to the lack of antibodies suitable for the detection of mature, cleaved IL-18 in macaque tissues.

We have considered several etiologic scenarios as possible explanations for lymphohistiocytic myocarditis in SIV-positive, CD8-depleted rhesus macaques after short-term treatment with RCV and PMPA, including: SIV infection, infection with opportunistic agents, IRIS and antiretroviral toxicity. We did not measure anti-desmin antibodies or pursue other methods to explore potential autoimmune mechanisms as a cause for these lesions. Since the SIV burdens in heart tissue were lower in the CART group macaques than the untreated animals, the animals were not clinically ill, and no opportunistic agents were identified, we believe that the myocarditis is due to the treatment with two NRTIs, resulting in cardiomyocyte injury and necrosis, subsequent mononuclear cell recruitment, lymphohistiocytic infiltration, and increased cardiac expression of TNF-α and CXCL9 in the affected animals. PMPA (Tenofovir or Viread©) manufactured by Gilead has been a part of many of the HAART regimens used to treat HIV-infected humans [56], [57], however, RCV has not been used for therapeutic purposes. Several studies have shown the association between treatment with NRTIs and cardiotoxicity and cardiovascular problems in humans. However, the toxicopathological impact of treatment with antiretroviral regimens containing multiple NRTIs has not been addressed in detail [30]. Prospective studies with the CD8-depleted, SIV/macaque model that include functional examination of the cardiovascular system will facilitate elucidation of the mechanism of myocardial toxicities that result from combined NRTI therapy.
